# Merging multimodal digital biomarkers into “Digital Neuro Fingerprints” for precision neurology in dementias: the promise of the right treatment for the right patient at the right time in the age of AI

**DOI:** 10.3389/fdgth.2025.1727707

**Published:** 2026-01-12

**Authors:** Ioannis Tarnanas, Azizi Seixas, Martin Wyss, Panagiotis Vlamos, Arzu Çöltekin

**Affiliations:** 1 Department of Psychiatry, Michigan Medicine, University of Michigan, Ann Arbor, MI, United States; 2 Global Brain Health Institute, Trinity College Dublin, Dublin, Ireland; 3 The Media and Innovation Lab, Department of Informatics and Health Data Sciences, University of Miami Miller School of Medicine, Miami, FL, United States; 4 Department of Psychiatry and Behavioral Health Sciences, University of Miami Miller School of Medicine, Miami, FL, United States; 5 Frost Institute for Data Science and Computing, University of Miami, Miami, FL, United States; 6 Bioinformatics and Human Electrophysiology Laboratory (BiHELab), Department of Informatics, Ionian University, Corfu, Greece; 7 Institute of Interactive Technologies, School of Computer Science, FHNW University of Applied Sciences and Arts Northwestern Switzerland, Brugg-Windisch, Switzerland

**Keywords:** Alzheimer's disease, biomarkers, dementia, digital biomarkers, digital health, precision medicine

## Abstract

Digital biomarkers are revolutionizing medicine in ways that were unimaginable a few years ago. Consequently, precision medicine approaches now realistically can promise personalization, i.e., the right treatments for the right patients at the right time, including earlier, targeted interventions which lead to a major paradigm shift in how medicine is practiced from reactive to preventive action. Although the scientific evidence is clear on the power of digital biomarkers, there is an unmet need for translating these findings into actionable insights in clinical practice. In this paper, we focus on Alzheimer's disease and related dementias (ADRD), and how digital biomarkers could empower clinical decision making in its preclinical stages. We argue that a new all-encompassing score is needed, akin to a BrainHealth Index linked to the established and validated risk stratifications frameworks and is directed at the prevention of ADRD. Specifically, we propose the new concept “Digital Neuro Fingerprint (DNF)”, built with simultaneous collection of multimodal digital biomarkers (speech, gait, eye movements etc.) from smartphone based augmented reality or virtual reality while an individual is immersed in activities of daily living. Fusing the captured multimodal digital biomarkers, data is automatically analyzed with custom combinations of machine- and deep-learning approaches and enhanced with explainable artificial intelligence (XAI) and uncertainty quantifications. We argue that DNF is useful for capturing ADRD progression and should supersede the biomarkers that are invasive and expensive to obtain, offering a sensitive and highly specific score that measures meaningful aspects of health for the patients in high-frequency intervals.

## 1 Introduction

Alzheimer's disease and related dementias (ADRD) are devastating neurodegenerative disorders and have long been recognized as heterogeneous conditions with diverse clinical presentations and underlying pathological mechanisms. ADRD are characterized by substantial heterogeneity across clinical, genetic, and neuropathological dimensions (1). A recent meta-analysis (2) identified three main sources of variability in ADRD: (1) risk factors e.g., age, sex, APOE genotype; (2) protective factors e.g., cognitive reserve, brain resilience, and resistance; and (3) co-occurring non-ADRD pathologies. These factors, along with amyloid (A) and tau (T) accumulation, may drive neurodegeneration (N) as outlined in the AT(N) framework (3). Evidence of neurofibrillary tangles (NFTs) in the association cortex with relative sparing of the hippocampus has been documented in some ADRD cases (4), highlighting the need to better define subtypes. This remains a central yet unresolved issue in the field, with significant implications for improving diagnostic precision and tailoring treatment strategies. Even though it has been subject to debate (5), recent research provides breakthrough evidence that distinct subtypes of ADRD exist, advancing our understanding of its complexity, and offering new avenues for diagnosis and treatment [e.g., (6–8)]. Most recently, bringing the subtype debate into proteomics, a proteomic analysis of over 1,000 cerebrospinal fluid (CSF) proteins in individuals with AD vs. healthy controls, has been performed using mass spectrometry (8), identifying five distinct AD subtypes. These correlated with distinct AD-related genetic risk factors, supporting that each CSF AD subtype reflects specific underlying molecular mechanisms. In parallel to the developments in medicine, new developments in computer science, specifically artificial intelligence (AI) based approaches have shown promise in differentiating AD subtypes. For example, deep learning models have been successfully leveraged (9) to classify the brain images of the AD patients from mild cognitive impairment (MCI), and cognitively normal (CN) ones based on to process magnetic resonance imaging (MRI), indicating that brain atrophy patterns at different stages of cognitive impairment and these can be captured to allow for a meaningful classification/subtyping.

Growing evidence supports the view that ADRDs are not a single condition, but part of a broader spectrum that ranges from subtle cognitive changes to severe impairment. Recent research suggests that there are potentially three to five distinct pathways, already in the early (MCI) stages. Our aim with this paper is to explore how the *integration* of multimodal markers obtained from brain imaging, blood biomarkers, and modern digital biomarkers from wearables, smartphones and extended (virtual, augmented, mixed) reality, powered by the latest developments in AI can create a major paradigm shift in neurology, ushering in a new era of precision medicine to effectively target the heterogeneity of ADRD physio-pathologies, for better research and clinical outcomes e.g., new drug developments or other effective treatments.

## 2 Current state of the art and the need for “Digital Neuro Fingerprints”

There are currently as many as 249 (mostly animal-based) research models for AD (10). While recent advancements in induced pluripotent stem cells (iPSCs) offer new ways to simulate AD (11, 12) many of the current models fall short in accurately representing the disease pathology due to inconsistencies with human studies and lack of agreement with the primary risk factor, aging. On the other hand, significant advancements have been made in the past three decades toward creating and verifying blood-based biomarkers for AD, which have been pivotal in shifting AD from a “conceptual” (i.e., clinician judgement) to a physio-biological framework (13). The amyloid-tau and neurodegeneration or neuronal injury, (AT(N)) blood-based classification system, introduced by the US National Institute on Aging (NIA) and the Alzheimer's Association (NIA-AA) research group in 2016, enhanced the study of AD by categorizing large patient groups (14). NIA-AA's categorization presents a unified way of thinking about how to group AD biomarkers at the individual level and provides an understandable and effective method for diagnosing AD, particularly beneficial for large-scale biological screening. However, while the AT(N) system holds pragmatic utility in diagnosing AD, its effectiveness in determining its stages, subtypes, and often-overlooked early/subtle cognitive and functional impairments have not been yet established.

Given the continuing need to improve ADRD diagnosis, staging, prognosis, and monitoring (15), there is broad consensus and abundant evidence that the non-invasive digital biomarkers and AI based approaches (deep/machine learning, i.e., DL/ML) can enhance ADRD evaluations in clinical settings and support clinical trials [e.g., (16–18)]. As an example, dozens of publications show that subtypes of ADRD can be identified by analyzing eye movements [e.g., (19–21)] and conversion from MCI to AD can be seen in composite digital biomarker data (22–25). Similarly, there are many studies on (digital) voice biomarkers, as both speech and tonality can contain information on cognitive impairments (22–25). Remarkably, “Subtype and Stage Inference (SuStaIn)”, an ML approach (26), is capable of identifying disease-specific phenotypes and their progression patterns from cross-sectional patient data. A recent study (27) utilized the SuStaIn algorithm (26) to develop a robust biological staging model for AD based on five biomarkers identified in the cerebrospinal fluid (CSF) (*Aβ42/40, pT217/T217, pT205/T205, MTBR-tau243*, and *non-phosphorylated mid-region tau*). The model was able to find a unique biomarker sequence, validated using samples from the BioFINDER-2 (*n* = 426) and Knight Alzheimer Disease Research Center (*n* = 222) cohorts. To characterize the molecular status of the participants utilizing the aforementioned model, the researchers demonstrated that individuals in CSF stages 2 and 3 (pT217/T217 and pT205/T205) were effectively classified as having Aβ positive and tau negative by positron emission tomography (PET) (A+T−). Conversely, individuals in CSF stages 4 (MTBR-tau243) or above exhibited positive results for amyloid-PET and tau-PET (A+T+), as per the AT(N) classification. Significantly, the CSF staging model also demonstrated prognostic usefulness. Initially, the researchers noted that individuals at various stages of cerebrospinal fluid (CSF) exhibited distinct rates of alteration in multiple biomarkers. As an example, the rates of Aβ accumulation across different CSF stages demonstrated the previously documented inverted U shape (28, 29). Specifically, participants at CSF stage 2 (pT217/T217) displayed the most significant rates of change. In contrast, the remaining imaging biomarkers and cognitive scores exhibited a higher rate of change as the CSF stages progressed, except for a plateau observed at the final stage, as anticipated (30, 31). This means that a model using a single CSF sample might be able to effectively find AD pathologies and give information about the stage of disease progression.

For subtyping, the neuroimaging data yields the most intriguing findings. Recent neuroimaging studies identified five distinct trajectories of brain atrophy progression for AD (32, 33). Analyzing 4,329 MRI scans from open-access MRI databases (34), monitored brain shrinkage in 3,262 AD/MCI vs. 2,944 CN participants through their entire lifespan (9 months to 94 years) and identified atrophy factors within various brain regions (medial temporal cortex, hippocampus, amygdala, striatum, thalamus, cerebellum, frontal cortex, parietal cortex, lateral temporal cortex, and lateral occipital cortex) using MRI imaging of AD patients combined with a mathematical framework from functional MRI brain networks. Consequently, the temporal pattern affected memory decline, and the cortical pattern connected to early onset and executive function impairment (34). However, the subcortical pattern showed the slowest progression of cognitive decline, often associated with APOE2. The model confirmed that changes in ADRD brains begin decades before symptom onset. In another key study (35), proposed a novel framework, called dimensional neuroimaging endophenotype (DNE), which analyzes neurobiological characteristics of neuropsychiatric and neurodegenerative disorders. The DNE condenses these complex traits into a digestible quantitative representation of brain phenotypes, serving as a reliable intermediate (i.e., endophenotype) to reflect genetic and etiological/causative factors. Several others employ ML algorithms to integrate data from neuroimaging, genetics, and clinical sources aiming to predict the onset and progression of cognitive and functional changes [e.g., (36–38)] or foresee cognitive impairments [e.g., (39)].

Examples highlighted above are only to illustrate an otherwise very rich body of research, and they are extremely encouraging. Given these developments, we posit that an overarching multimodal approach that integrates and personalizes digital biomarkers, i.e., our proposed “Digital Neuro Fingerprints” concept will advance the current state of the art.

## 3 Possible directions to accelerate adoption of digital biomarkers in research and in clinical practice

Despite the rigorous efforts outlined above, the potential etiological connections between *the right treatment for the right patient at the right time* and various trajectories of MCI across diverse cultural populations are yet to be established using AI/ML. Advanced machine learning (ML) algorithms, especially those based on neural networks, can be difficult to understand and traceback (40). Many cannot explain their predictions e.g., of transition from MCI to dementia, creating trust issues due to lack of explainability (41). Emerging research fields called explainable artificial intelligence (XAI) and Uncertainty Quantification (UQ) address these trust issues (42). While many XAI efforts visualize the steps of the algorithm and the decision space [e.g., (43, 44)] a proper XAI effort necessitates more than a technique, i.e., a critical reflection on the representativeness of the training data, and thus globally-collected, affordable, longitudinal, and multimodal data. On the other hand, UQ relies on cycle consistency, statistically evaluates uncertainties related to neural network outputs, detects input data corruption and distribution shifts, and enhances neural network dependability for disease prognostication. Integrating XAI/UQ into ML/DL models improves confidence in predictions, enhances usability of XAI rules, and clarifies the modeling process. We argue that implementing XAI with UQ could have an impact on enhancing specificity within a digital biomarker-driven classification system for MCI and AD, aligning with the AT(N) classification. Another reason to utilize AI/ML with multimodal digital biomarker data is that this can be achieved on an off-the-shelf device such as a smartphone, which we believe might contribute towards closing the global socio-economic gaps and enable a more accessible and frequent diagnosis and monitoring of AD subtypes. Our prior work (45–48) demonstrates the utility of AI-based persuasive technologies for clinical trial engagement, precision health education, and predictive modeling in chronic disease and neurocognitive impairment. In the PREDHICT, DORMIR, and UDECIDE projects (45, 47, 48), we showed that culturally sensitive, AI-driven systems can improve trust, literacy, and adherence in underserved communities. Our recent publication (45) further provides a new taxonomy for equitable digital access—crucial for ensuring real-world uptake (49).

## 4 From concept to practice: the DNF toolbox—a multimodal digital biomarker solution

**Figure 1:**
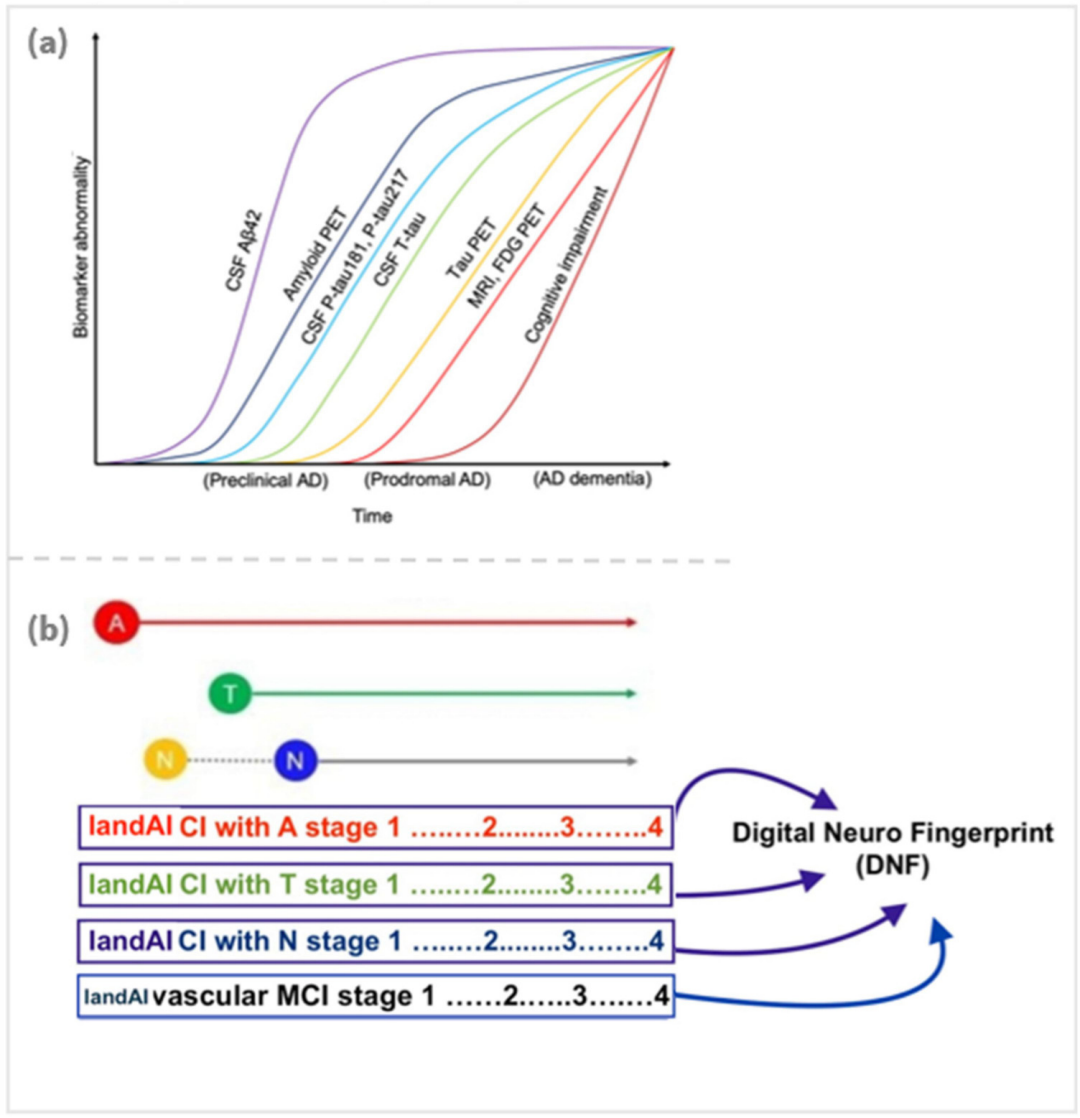
**(a)** Biomarker abnormality over time. Reproduced from “CSF tau biomarkers as statistical mediators of the relationship between Amyloid PET and Tau PET (**E**) A model synthesizing the findings in this study, together with previous literature (*37*), indicating an approximative ordering of how different measures change during the disease course. The results in this study suggest that changes in CSF P-tau181 and P-tau217 may start shortly after Amyloid PET. In parallel, or shortly thereafter, CSF T-tau may increase. Both CSF P-tau and T-tau markers start to change before significant uptake is detected by Tau PET. Approximate overall disease stages (preclinical, prodromal, and dementia) are indicated on the *x* axis. We acknowledge that there may be large interindividual differences in the timing of different events (especially changes in cognition) due to individual reserve and vulnerability factors that may modulate the relationships between different disease hallmarks. FDG, fluorodeoxyglucose” by Mattsson-Carlgren et al., licensed under CC BY-NC 4.0. **(b)** A possible model connecting the biological classification of AD to unique DNS digital biomarkers and creating a unique proxy: Digital Neuro Fingerprint (DNF).

Inspired by the DNF concept, a novel platform is being developed merging a proprietary smartphone app that simulates complex activities of daily living, while users verbally describe their steps, with *IandAI*, a generative AI driven “brain digital twin” to detect ADRD via real-world data (voice, eye tracking, gait, sleep, facial cues, typing). Probabilistic risk scores, created using ML algorithms undergirded by Bayesian statistics, refer to distinct digital phenotypes representing disease stage, subtypes, and progression, also leveraging neuropsychological, biological, and brain pathology tests. Thus, DNF toolbox uniquely encompasses the capacity to objectively assess indicators of cognition and function, including visuospatial exploration behaviors, perceptual motor coordination, gait behavior, and their joint micro-changes (50, 51) including voice biomarkers. These probabilistic risk scores precisely align with the AT(N) classification creating unique “DNFs” (Figure 1).

Cost-effective and high-frequency tests enabled by active digital biomarkers can create detailed “*longitudinal trajectories*” that can assist in disease staging and subtyping. Employing functional mixed-effects linear models to examine temporal changes in DNF phenotypes and integrating UQ addresses XAI needs and identify non-linear biomarker associations. This is especially important for proxies with high specificity in complex neurodegenerative disorders and should inform future research aimed at a deeper understanding of growing digital biomarker evidence. A deeper elaboration of the DNF model index and its notation are provided in the Supplementary Material.

### 4.1 Best of both worlds: combining the AT(N) classification system with DNF for diagnosis, prevention and therapy management for AD

We contend that optimal clinical proxies should be specific to the characteristic of interest and not linked to unrelated phenotypes. For example, the SensorLM framework from Google Research (2024) shows how large language models can capture multimodal wearable signals in a transparent way, which resonates with our vision for DNFs (53). Digitally tracking actual behaviors with high ecological validity, such as shifts in navigation and visual exploration, show high specificity for early AD stages. These changes are typically characterized by amyloid and tau pathology preceding deficits in other areas such as episodic memory and can be detected during the initial Braak pathological staging [e.g., (54–57)]. Also importantly, studied correlations between motor decline and dementia pathology, found that macroinfarcts, not tau tangles, were linked to initial gait function decrease prior to cognitive impairment, suggesting a need to explore gait impairment as a precursor for vascular cognitive impairment (58). Given that mixed pathology is prevalent (59), e.g., AD and vascular diseases frequently co-occur, such findings could have broader implications for predicting subtle cognitive and functional impairments. Details of a target simulation, including the list of digital biomarkers included in the study, is provided in the Supplementary Material.

**Figure 2:**
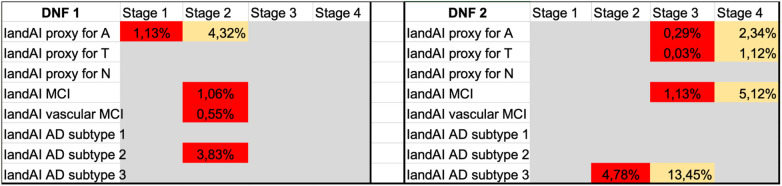
Hypothetical visualization of two different DNFs (DNF 1 and DNF 2) corresponding to two different patients. DNF 1 is a proxy for stage 1 AD-related pathophysiological changes—the amyloid-β pathway (“A”), MCI and stage 2 probable vascular damage, mostly corresponding to AD subtype 2 based on dimensional neuroimaging endophenotypes (DNE). In addition, in each box, there are values like for example in the IandAI proxy for A: “1.13%”. This means that the probability of the transition to the next stage within 1 month is 13%. As a worked example, a value of 0.55% should be interpreted as: Immediate risk of rapid decline (cognitive and functional) within less than a month with 55% probability. For example, that would be an imminent transition event passing the threshold of what is characteristic of Stage 2 and predicting that the said patient phenotype is presenting manifestations that will rapidly transition to Stage 3 based on the trajectory of historic values. DNF 2 is a proxy for stage 3 AD-related pathophysiological changes—the amyloid-β pathway (“A”), tau pathology (“T”), MCI and stage 1–2 probable vascular damage, mostly corresponding to AD subtype 3. Finally, the red color would indicate “high risk,” and the probability inside is the uncertainty quantification for the AI prediction. The yellow color indicates “medium risk” and the probability of the transition to the next state within a set period of time, e.g., 24 months, and the probability inside is the uncertainty quantification for the AI prediction. Grey would indicate “no risk”. Historically “high-risk” categories transition from Stage to Stage within a >12-month time window. This is aligned with other models predicting 6–8 months for examining progressions, e.g., ADNI or UK biobank (also see Supplementary Material). Stage progression values should be interpreted as the imminent risk of hospitalizations. For entries that appear under stage 4 (2.34%), i.e., the particular values are informative for healthcare providers and a validated prediction there can add clinical value for prioritizing “high-risk for hospitalization” patients, thus fast tracking the said patients and increasing efficiency at the diagnostic clinic flow.

To explain how DNFs are relevant in clinical settings, and how they might be useful in the future for AD research and therapy, we created the hypothetical visualization in Figure 2, designed to take into account optimal treatment options for *the right patient, at the right moment*.

The primary benefit of DNFs is that they can be produced with simple digital measurement that captures multimodal data, while XAI and UQ provide immediate data and process quality validation. At this stage of this on-going project, we propose a two-step process to incorporate plasma p-tau217 with validated components of the DNF concept.

Several others suggest a two-step process for identifying amyloid-β (Aβ) positivity in individuals with cognitive impairment (60, 61), [e.g., (62)] propose an initial blood-based model to classify patients into *low-*, *medium-*, or *high-*risk groups for Aβ-PET positivity. In Step-2, only moderate-risk patients would undergo CSF Aβ42/Aβ40 testing. Depending on thresholds in Step-1, 88.2%, 90.5%, and 92.0% accuracy was obtained in detecting Aβ-PET status, and reduced CSF tests by 85.9%, 72.7%, and 61.2% respectively (62).

In our model, step two would utilize the DNF MCI with a *high amyloid likelihood score* (once FDA-regulated as an adjunct diagnostic) and further confirmatory testing would be ordered only in uncertain cases. The DNF alternative provides a financially attractive approach for identifying AD, especially in geographically isolated regions and beyond the confines of memory clinics. For prevention, integrating blood biomarkers with e.g., monthly time-series DNF sub-scores could effectively categorize risk (low, middle, high) as a monitoring/predictive biomarker. The FDA-NIH's BEST (63) defines predictive biomarkers as those indicating the likelihood of an outcome post-drug exposure based on presence or changes in the biomarker. To validate a biomarker, rigorous clinical research is required, usually involving random assignments to varied treatments (or a placebo baseline) irrespective of a biomarker's presence or absence. Demonstrating a biomarker's predictability is challenging, but if successful, could reduce the need for confirmatory (CSF) or positron emission tomography (PET) testing for most patients.

Incorporating comprehensive data on biological trajectories and significant digital brain health phenotypes, such as motor and subtle cognitive and functional trajectories, can predict the precise timeline of dementia onset and inform treatment decisions. Utilizing XAI to correlate AD biomarkers with early cognitive, behavioral, and functional characteristics shows potential in aiding comprehensive biological screening and decision-making in diagnostics and therapy, providing a globally accessible, sustainable solution for AD patients' future.

## 4 Conclusions

Integrating DNFs and advanced technologies like XAI and UQ could revolutionize ADRD research and clinical practice, paving the way for truly *personalized* treatments as DNFs potentially offer a single but potent digital score significantly simplifying clinical decision support. Our proposed two-step staging workflow, incorporating plasma p-tau217 with validated DNFs, enhances diagnostic accuracy while reducing invasive testing. The use of a two-step process for detecting amyloid-β (Aβ) positivity provides a practical screening method, especially relevant with emerging anti-Aβ immunotherapies for ADRD. Looking ahead, integrating blood biomarkers with time-series DNF sub-scores offers a promising avenue for monitoring and predicting ADRD risk, which could be especially beneficial in remote areas and those with limited resources. Identifying and validating predictive biomarkers, requiring rigorous clinical methodologies, is vital for the utility of DNFs in clinical practice. Comprehensive data on biological trajectories and digital phenotypes holds transformative potential in predicting dementia progression and guide treatment decisions. By harnessing XAI to elucidate correlations between biomarkers and early cognitive and functional characteristics, DNFs offer a novel framework for precise screening and decision-making in ADRD diagnosis and therapy.

In summary, the application of DNFs represents a paradigm shift in the approach to ADRD research and clinical care, offering personalized, efficient, and globally accessible solutions. Continued interdisciplinary collaboration and rigorous validation efforts are essential to realizing the full potential of DNFs in improving outcomes for ADRD patients worldwide.

## Data Availability

The original contributions presented in the study are included in the article/Supplementary Material, further inquiries can be directed to the corresponding author.
