# Supplementary material for: Merging multimodal digital biomarkers into “Digital Neuro Fingerprints” for precision neurology in dementias: the promise of the right treatment for the right patient at the right time in the age of AI
Source: Front Digit Health. 2026 Jan 12;7:1727707. doi: 10.3389/fdgth.2025.1727707 (PMC12832889; doi:10.3389/fdgth.2025.1727707)
Supplement: Supplementary file 1 [file Datasheet1.pdf]

Supplementary Material

Merging multimodal digital biomarkers into “Digital Neuro Fingerprints” for precision neurology in dementias: The promise of the right treatment for the right patient at the right time in the age of AI

Ioannis Tarnanas[1,2,3], Azizi Seixas[3,4,5], Martin Wyss[6], Panagiotis Vlamos[6], Arzu Çöltekin[7*]

[1]Department of Psychiatry, Michigan Medicine, University of Michigan, Ann Arbor, USA

[2]Global Brain Health Institute, Trinity College Dublin, Dublin, Ireland

[3]The Media and Innovation Lab, Department of Informatics and Health Data Sciences, University of Miami Miller School of Medicine, Miami, FL, United States

[4]Department of Psychiatry and Behavioral Health Sciences, University of Miami Miller School of Medicine, Miami, FL, United States

[5]Frost Institute for Data Science and Computing, University of Miami, Miami, FL, United States

[6]Bioinformatics and Human Electrophysiology Laboratory (BiHELab), Department of Informatics, Ionian University, Corfu, Greece.

[7] Institute of Interactive Technologies, FHNW University of Applied Sciences and Arts Northwestern Switzerland, Brugg-Windisch, Switzerland

*** Correspondence:** arzu.coltekin@fhnw.ch

1 The DNF model index to predict stage and decline in dementia: notation for the multimodal markers

The question we address is to predict stage and decline in dementia within the scope of accelerating clinical trials. To do so, we are introducing the concept of DNF as a single posterior probability of “high-risk status,” e.g. prodromal Alzheimer’s / neurodegeneration / cognitive decline within 24 months. We will call that latent event D=1 (high risk of progressing), and D=0 otherwise.

> **I: imaging markers (MRI, PET, etc.)** e.g. hippocampal volume Z-score, cortical thinning in temporo-parietal regions, amyloid/tau PET load, FDG-PET hypometabolism pattern. Structural+functional imaging combinations like these have been modeled jointly in Bayesian multimodal neuroimaging frameworks to separate late-life depression from controls and detect “neuromarkers,” showing that information can be shared across modalities in a probabilistic way (Bhaumik et al., 2023).

B: biochemical / blood-based biomarkers e.g. plasma p-tau181 / p-tau217, Aβ42/40 ratio, NfL (neurofilament light), GFAP. Blood biomarker panels have recently been shown to predict brain amyloid status and stratify Alzheimer's pathology almost at PET/CSF-level performance (Christodoulou et al., 2025).

D: digital phenotyping features from wearables / smartphones e.g. gait variability from inertial sensors, turn hesitation, navigation/orientation performance in an augmented reality wayfinding task, speech timing, facial cues analysis and speech cadence, while describing the steps taken for the navigation/orientation task. Also independently validated smartphone-derived mobility/cognition patterns have already been linked to mild cognitive impairment and dementia risk, and have been used to classify preclinical AD (Muurling et al., 2023).

We will assume we observe one subject with DNF measurements: (I,B,D) with the goal to estimate P (D=1 ∣ I,B,D), which is interpretable as "their individualized posterior risk."

We then define the posterior log-odds:

$$\log[P(D=1 \mid \mathbf{I},\mathbf{B},\mathbf{D}) / P(D=0 \mid \mathbf{I},\mathbf{B},\mathbf{D})] =$$
$$\log[P(D=1)/P(D=0)] +$$
$$\log[P(\mathbf{I}|D=1)/P(\mathbf{I}|D=0)] +$$
$$\log[P(\mathbf{B}|D=1)/P(\mathbf{B}|D=0)] +$$
$$\log[P(\mathbf{D}|D=1)/P(\mathbf{D}|D=0)]$$

We call each log-likelihood ratio a modality-specific risk contribution. $\Delta\_\mathbf{I}$, $\Delta\_\mathbf{B}$, $\Delta\_\mathbf{D}$ = log-likelihood ratios (Bayes factors). For example, plasma p-tau and NfL can already predict amyloid PET positivity and near-term progression, so this term captures that biochemical "AT(N)" signal without needing invasive CSF. Similarly, for the digital biomarker contribution, abnormal gait micro-variability, disorientation during real-world wayfinding captured by the augmented reality task, speech pause metrics, reduced spontaneous facial expressivity (particularly facial micro-movement complexity) and cognitive slowing in voice interaction data, which have already been linked to early cognitive decline and subjective cognitive decline status in older adults.

Then the prior term is set, which can encode age, APOE4, family history, vascular burden, education, etc., or simply be set from epidemiological base rates in the intended-use population:

$$\text{logit}[P(D=1 \mid \mathbf{I},\mathbf{B},\mathbf{D})] = \Delta\_\text{prior} + \Delta\_\mathbf{I} + \Delta\_\mathbf{B} + \Delta\_\mathbf{D}$$

Finally, DNF as individualized posterior probability of near-term progression can be described as:

$$P(D=1 \mid \mathbf{I},\mathbf{B},\mathbf{D}) = \sigma(\Delta\_\text{prior} + \Delta\_\mathbf{I} + \Delta\_\mathbf{B} + \Delta\_\mathbf{D})$$

We thus calculate DNFs with a strict Bayesian fusion formula. Each Δ term acts as a modality-specific Bayes factor:

- Δ_**I**: neuroimaging evidence (atrophy, hypometabolism, amyloid/tau burden)
- Δ_**B**: biochemical proof (p-tau, NfL, Aβ ratio)
- Δ_**D** : digital evidence (mobility, voice, cognitive-motor metrics)

This formulation of DNF naturally supports longitudinal updating. As we collect new smartphone data streams actively and passively (e.g. gait slowing over 3 months, voice rate slowing and navigation errors increasing), we just recompute ΔD with the new D and update the posterior without re-running an MRI or another blood draw. Continuous remote updating of risk has been highlighted as a core value proposition of digital biomarkers for neurodegenerative disease, fatigue in neuro conditions, and cognitive frailty. Also, it decomposes individual risk into interpretable "where is the risk coming from," which is critical for explainability requirements in clinical AI and SaMD regulatory review. Clinical decision support models for Alzheimer's and related dementias are under pressure to justify predictions to clinicians, not just output a score.

2 Digital biomarkers and targeted simulation study

Digital biomarkers that measure psychomotor speed, ecological function, and facial affect were already as good as MRI, but combining them with imaging and molecular data made a big difference in the clinic. Compared with the UK Biobank MRI-only benchmark (AUC ≈ 0.73), our multimodal fusion nearly closed the gap between population screening and clinical cohorts. In summary, combining structural, molecular, and digital markers within a Bayesian framework achieved state-of-the-art discrimination (AUC 0.92) and offers a scalable, explainable approach for early detection of cognitive decline. Table 1 below lists those digital biomarkers.

Table 1: List of digital biomarkers:

Data Modality	Type of biomarker	Data collected
Facial cues analysis	digital	Facial Action Unit (AU) intensity distributions<br>Emotional reactivity entropy<br>Facial asymmetry index<br>Response latency to affective stimuli<br>Blink/micro-expression frequency<br>Dynamic range of affect (temporal variance of AUs)
Micro-movements analysis	digital	a) Micro-motor and gait exercises, b) AR based complex activity of daily living (navigation/orientation assessment), c) speech assessment task

We carried out a targeted simulation study using parameters derived from the Alzheimer's Disease Neuroimaging Initiative (ADNI) to estimate how well DNFs discriminate between individuals who remain stable and those who progress over a 24-month period. We also compared these results to analogous findings from the UK Biobank to provide broader context. In more detail, we performed a Monte Carlo simulation (1,000 replicates) using ADNI parameters to evaluate the contributions of imaging (I), blood (B), and digital/functional (D) biomarkers in predicting cognitive decline over two years. We used the DNF Bayesian fusion model to figure out the posterior conversion probabilities for each modality, which was modelled as a class-conditional Gaussian. Mean AUCs (95 % CI): imaging 0.81 (0.78–0.83), blood 0.75 (0.72–0.78), digital 0.84 (0.81–0.86), imaging + blood 0.86, imaging + digital 0.91, blood + digital 0.87, and full fusion 0.92 (0.90–0.93). So, Bayesian fusion improved discrimination by +0.11 AUC over imaging alone and +0.08 over digital alone, which shows that each method gives different information. The model remained robust under variable prevalence, feature correlation, and 30 % missingness.

In summary, combining structural, molecular, and digital markers within a Bayesian framework achieved state-of-the-art discrimination (AUC 0.92) and offers a scalable, explainable approach for early detection of cognitive decline.

3 How the simulation was run

We ran 1,000 Monte-Carlo replicates. In each replicate:

1. Outcomes: We simulated 300 individuals with a 20% probability of conversion.
2. Features: For each subject, imaging, blood, and digital feature vectors were drawn from the class-specific Gaussian distributions estimated from ADNI data.
3. Models: We computed risk probabilities for seven model configurations—each single modality, every pairwise combination, and the full tri-modal fusion.
4. Evaluation: AUCs were calculated using DeLong's method, and we summarized mean AUCs with 95% confidence intervals (CIs) across all replicates.

We also performed sensitivity analyses to confirm that results were robust to variations in disease prevalence, inter-feature correlations, and missing data.

A. Simulation results

The table below summarizes the mean AUCs across replicates:

Model	Mean AUC (95% CI)	Key insight
Imaging only (I)	0.81 (0.78–0.83)	MRI and PET markers of atrophy and hypometabolism.
Blood only (B)	0.75 (0.72–0.78)	p-tau, Aβ42/40 ratio, NfL.
Digital/Functional only (D)**	0.84 (0.81–0.86)	High-resolution behavioral, cognitive, and affective measures.
Imaging + Blood (I + B)	0.86 (0.84–0.88)	Combines neurodegeneration with molecular pathology.
Imaging + Digital (I + D)	0.91 (0.89–0.92)	Marries brain structure with real-world cognitive/affective function.
Blood + Digital (B + D)	0.87 (0.85–0.89)	Pathology plus ecological behavior.
Imaging + Blood + Digital (I + B + D)**	0.92 (0.90–0.93)	Full multimodal fusion.

The corrected AUC for the digital/functional modality (0.84) shows that modern digital biomarkers can already rival the discriminative performance of MRI alone. However, the Bayesian fusion still provided a meaningful improvement in prediction.

- Compared with imaging alone: AUC increased from 0.81 to 0.92 (+0.11 absolute gain).
- Compared with digital alone: AUC increased from 0.84 to 0.92 (+0.08 absolute gain).
- Compared with blood alone: AUC improved by +0.17.

Even when digital biomarkers are highly informative on their own, they do not fully replace the biological insight provided by imaging and fluid measures. Each domain contributes something distinct:

- Imaging captures the structural and metabolic footprint of neurodegeneration.
- Blood biomarkers provide early molecular evidence of tau, amyloid, and axonal injury.
- Digital and functional measures capture the lived experience of cognitive and emotional change, including subtle affective flattening, slowed reaction time, or reduced facial expressivity.

When these signals are combined probabilistically, the model essentially estimates a “posterior disease probability” that integrates molecular, structural, and behavioral evidence. The resulting AUC of 0.92 is consistent with the best multimodal fusion models reported in the ADNI literature for short-term conversion prediction.

B. Comparison with UK Biobank data

To situate these findings in a broader context, we compared them with results from the UK Biobank, which includes over 40,000 participants with MRI and cognitive testing but currently lacks molecular and fine-grained digital biomarkers. A recent UK Biobank analysis that classified “Positive-Agers” versus “Cognitive Decliners” using MRI and demographic variables achieved an AUC of approximately 0.73. The lower performance likely reflects both the healthier, population-based nature of the sample and the absence of molecular and behavioral modalities. If UK Biobank-style models were extended to include plasma biomarkers and digital/functional data of the type modeled here, our simulations suggest that AUC could increase into the 0.85–0.88 range, effectively bridging most of the gap between population screening and clinic-based prediction performance.

4 Sensitivity and robustness

We examined several robustness factors:

- Missing data: When up to 30% of subjects lacked one modality, the model’s AUC dropped by less than 0.02 points because the Bayesian framework naturally re-weights available evidence.
- Prevalence: AUC remained stable across base conversion rates from 10% to 30%, although predictive values adjusted as expected.
- Correlations: Moderate inter-feature correlations ($r \approx 0.4$–0.6) did not materially affect discrimination, supporting the conditional-independence assumption used in the model.

These checks indicate that the observed performance is not a fragile artifact of parameter choices.

References used in this supplement

Bhaumik, D.K., Wang, Y., Yen, P.-S., Ajilore, O.A., 2023. Development of a Bayesian multimodal model to detect biomarkers in neuroimaging studies. Front. Neuroimaging 2, 1147508. https://doi.org/10.3389/fnimg.2023.1147508

Christodoulou, R.C., Vamvouras, G., Sarquis, M.D., Petrou, V., Papageorgiou, P.S., Rivera, L., Morales, C., Rivera, G., Vassiliou, E., Solomou, E.E., Papageorgiou, S.G., 2025. Magnetic Resonance Imaging and Cerebrospinal Fluid Biomarker Clustering Defines Biological Subtypes of Alzheimer’s Disease. Biomedicines 13, 2632. https://doi.org/10.3390/biomedicines13112632

Muurling, M., De Boer, C., Vairavan, S., Harms, R.L., Chadha, A.S., Tarnanas, I., Luis, E.V., Religa, D., Gjestsen, M.T., Galluzzi, S., Ibarria Sala, M., Koychev, I., Hausner, L., Gkioka, M., Aarsland, D., Visser, P.J., Brem, A.-K., 2023. Augmented reality versus standard tests to assess cognition and function in early Alzheimer’s disease. Npj Digit. Med. 6, 234. https://doi.org/10.1038/s41746-023-00978-6
